# Lipid Disorders Management Strategies (2024) in Prediabetic and Diabetic Patients

**DOI:** 10.3390/ph17020219

**Published:** 2024-02-07

**Authors:** Laura Gaita, Bogdan Timar, Romulus Timar, Zlatko Fras, Dan Gaita, Maciej Banach

**Affiliations:** 1 Second Department of Internal Medicine, “Victor Babes” University of Medicine and Pharmacy, 300041 Timisoara, Romania; 2 “Pius Brinzeu” Emergency Hospital, 300723 Timisoara, Romania; 3 Department of Vascular Disease, University Medical Center Ljubljana, 1000 Ljubljana, Slovenia; 4 Faculty of Medicine, University of Ljubljana, 1000 Ljubljana, Slovenia; 5 Department of Cardiology, “Victor Babes” University of Medicine and Pharmacy, 300041 Timisoara, Romania; 6 Institutul de Boli Cardiovasculare, 300310 Timisoara, Romania; 7 Department of Preventive Cardiology and Lipidology, Medical University of Lodz (MUL), 90-419 Lodz, Poland; 8 Department of Cardiology and Congenital Heart Disease of Adults, Polish Mother’s Memorial Hospital Research Institute (PMMHRI), 93-338 Lodz, Poland; 9 Cardiovascular Research Centre, University of Zielona Gora, 65-417 Zielona Gora, Poland

**Keywords:** atherogenic dyslipidaemia, diabetes mellitus, prediabetes, cardiovascular risk, treatment targets, LDLc, non-HDLc, lipid-lowering pharmacologic agents

## Abstract

Dyslipidaemia is a modifiable risk factor commonly associated with diabetes mellitus and prediabetes, with a major impact on the early development of atherosclerotic cardiovascular disease. Various studies have tried to identify the key treatment targets, their optimal values according to patients’ CV risk, and the most efficient yet safe therapeutic agents which, alongside lifestyle changes, would improve lipid levels and reduce cardiovascular mortality and morbidity. Currently, there are multiple pharmacologic options that can be used in the management of dyslipidaemia, such as statins, ezetimibe, bempedoic acid, PCSK9 inhibitors, n-3 polyunsaturated fatty acids or fibrates, to name only a few, while many other are under development. In the current setting of a continuously increasing population of patients with metabolic disorders, this review aims to summarise current knowledge regarding lipid disorders and the recommendations of recent guidelines in treating dyslipidaemia in patients with diabetes mellitus or prediabetes.

## 1. Introduction

Dyslipidaemia is a key modifiable risk factor for cardiovascular disease (CVD) and premature death. The importance of a comprehensive approach to lipid disorders is derived from the major impact of CV health in this era of noncommunicable diseases, with CVD—mainly atherosclerotic CVD—being the leading cause of death, globally [1]. Even more so, in patients with diabetes mellitus (DM), the risk of CV disease is increased 2–4 times in comparison to patients without diabetes [2]. In prediabetes—defined either through increased fasting, postprandial or 2 h oral glucose-tolerance test glycaemic levels or through an increased haemoglobin A1c (HbA1c)—although the data is scarcer than for DM, the risk of CVD is higher than in individuals with a normal glucose metabolism [3].

Lipid disorders—quantitative or qualitative disturbances of plasmatic lipoproteins caused by either their excessive production or defective metabolism—are, themselves, some of the most common noncommunicable diseases and, from another point of view, can be considered an ever-expanding topic of research, with continuous discoveries of new genetic or molecular causes or mechanisms of action that contribute to the development of innovative classes of new pharmacologic agents [4].

The prevalence of dyslipidaemia is different across geographic regions, in different populations, and, as expected, with different definitions of dyslipidaemia itself, with a prevalence of 20–60% reported by multiple publications in the case of hypercholesterolemia. Moreover, approximately half of the persons who had a total cholesterol level higher than 5.17 mmol/L (200 mg/dL) were not aware of this metabolic disorder, with implications regarding CV prevention [5,6,7]. The prevalence of dyslipidaemia is even more increased in patients with obesity, namely 60–80%, or even over 80% in the presence of other CV risk factors [8]. In DM, the prevalence of dyslipidaemia varies across studies, with values from 60% to over 90% of patients, while in prediabetes, although values are even more diverse, the prevalence is higher than in the general population [9,10,11,12,13].

A specific type of lipid disorder, usually associated with obesity, metabolic syndrome, type 2 DM or prediabetes, is the atherogenic dyslipidaemia, characterised by increased plasmatic levels of both fasting and postprandial triglycerides (TGs), low levels of high-density lipoprotein cholesterol (HDLc) and apolipoprotein A1 (ApoA1), the increase of small, dense low-density lipoprotein (LDL) particles and of the lipoproteins that contain apolipoprotein B (apo B), parameters that have been independently associated with a higher CV risk [14,15]. One of the key mechanisms that are involved in the development of atherogenic dyslipidaemia is insulin resistance which contributes to an increased release of free fatty acids from the adipocytes, with their subsequent liver uptake and an increased synthesis of very low-density lipoproteins (VLDL), especially VLDL-1, large lipoproteins, rich in TGs that will lead to an increased production of small, dense LDL particles and a decreased HDLc. This type of dyslipidaemia has a particularly high impact on the development of CVD since, although all LDL particles are proven to be atherogenic, the small, dense ones are even more so due to their increased ability to pass through the arterial intima, their lower affinity for the LDL receptor and their reduced antioxidant properties in comparison to other types of particles [14,15,16,17].

Although the association between dyslipidaemia and the development of atherosclerosis and, consequently, the increased risk of CVD is now well known, it was proven only in 1961 with the results of the Framingham Study, which has additionally shown a connection between hypertension and left ventricular hypertrophy and CVD, and that has led to the newly defined notion of CV risk factor [18]. Nevertheless, although multiple parameters such as blood pressure, body mass index (BMI) or blood glucose levels have a U-shaped curve in relation to CV risk, this is not the case for LDLc, which has a linear association with CV risk, according to multiple studies. It has been suggested that a 0.77 mmol/L (30 mg/dL) reduction in LDLc contributes to a decrease in coronary heart disease of approximately 30% and that this reduction is even more important if a lower level of LDLc is achieved, including levels previously considered to be too low, such as 0.77–1 mmol/L (30–40 mg/dL) or even less, which are still extremely safe. Moreover, the progression of atherosclerosis is stopped with an LDLc below 1.8 mmol/L (70 mg/dL), with proofs of reduction in the volume of the atherosclerotic plaque if the LDLc is lower, supporting the principle that, regarding LDLc, “the lower the better” [19,20,21,22,23,24]. Nevertheless, another word should be added to the principle, “the earlier and the lower the better”, since LDLc has a cumulative effect on the progression of atherosclerosis, with an apparent threshold of 5000 mg-years that is associated with a significant increase in the risk of developing an acute myocardial infarction [25].

## 2. Therapeutic Targets

Taking into consideration the previously mentioned association between LDLc and atherosclerotic CVD, the guidelines’ recommendations for the total cholesterol and LDLc targets have changed in the past few years. The European Society of Cardiology (ESC) has updated the therapeutic targets in their *2019 Guidelines for the management of dyslipidaemias*. In order to achieve the primary or secondary prevention of CVD, with an optimal risk–benefit ratio of the recommended lifestyle and pharmacologic interventions, the treatment targets in dyslipidaemias are chosen in relation to the CV risk of the patient.

To assess the CV risk itself, the ESC has proposed two new tools in the *2021 Guidelines on cardiovascular disease prevention in clinical practice*, namely the SCORE2 and SCORE2-OP, based on complex data sets, representative of the population of Europe, with different versions for the different countries of the continent, grouped into four categories according to the countries’ risks. These tools estimate the risk of developing a fatal or non-fatal CVD in 10 years and are recommended especially in apparently healthy individuals, without documented CVD. The charts take into consideration the country, gender, age, smoking status, systolic blood pressure and level of non-HDLc, the latter being different to previous guidelines, which included the total cholesterol level [26,27]. The switch from total cholesterol to non-HDLc—and the gradual inclusion of apoB in the standard lipid panel assessment—is explained by multiple studies that have shown the impact of triglyceride-rich lipoproteins, still atherogenic, on the development of ASCVD, especially in patients with obesity, metabolic syndrome, prediabetes or type 2 DM, diseases in which the assessment of the LDLc on its own could underestimate the impact of the lipid imbalance or the impact of the treatment in the primary- or secondary-CV prevention [27,28].

In 2023, the *ESC Guidelines for the management of cardiovascular disease in patients with diabetes* has proposed a new tool for assessing CV risk in patients with type 2 DM over the age of 40 years, the SCORE2-Diabetes, a 10-year CVD risk model for individuals with this condition, which includes, alongside the previously mentioned parameters, three new ones: the age at diabetes diagnosis, the HbA1c and the estimated glomerular filtration rate (eGFR). This tool should be used in the absence of atherosclerotic CVD (ASCVD) or severe target-organ damage, and groups patients into four categories—very high risk (if the scoring is ≥20%), high risk (10–20%), moderate risk (5–10%) or low risk (<5%). However, if a patient with T2DM has either an established ASCVD or severe organ damage—defined as eGFR < 45 mL/min/1.73 m^2^, or an eGFR 45–59 mL/min/1.73 m^2^ and microalbuminuria (urinary albumin/creatinine ratio—UACR 3–30 mg/mmol or 30–300 mg/g), or macroalbuminuria (UACR > 30 mg/mmol or 300 mg/g), or presence of microvascular disease in at least three different sites—then the CV risk is already considered to be very high [29]. All the tools recommended by the European Society of Cardiology in order to assess the CV risk can be found on the ESC CVD Risk Calculation App (https://www.escardio.org/Education/ESC-Prevention-of-CVD-Programme/Risk-assessment/esc-cvd-risk-calculation-app (accessed on 3 February 2024)).

The CV risk assessment is, however, more difficult in patients with prediabetes, since this condition is not taken into consideration in any of the currently used tools. Nevertheless, the SCORE2 and SCORE2-OP can be used in this population, while retaining the thought that, probably, the real CV risk is higher than the one predicted [27].

Regarding the lipid-related therapeutic targets, the primary target is represented by the LDLc, the secondary (sometimes also named co-primary) targets are the non-HDLc and, according to some authors, apoB, while other parameters that are taken into consideration are the TGs, HDLc and lipoprotein (a) (Lp(a)). The LDLc target is <1.4 mmol/L (55 mg/dL) and a reduction of at least 50% compared to the initial level (whichever of the two is the lowest) in patients with a very high CV risk, <1.8 mmol/L (70 mg/dL) and a reduction of at least 50% compared to the initial level (whichever of the two is the lowest) in patients with a high CV risk and <2.6 mmol/L (100 mg/dL) in patients with a moderate CV risk; in patients with a low CV risk, no clear recommendations can be given in patients with T2DM, although the target can be considered to be <3 mmol/L (116 mg/dL) in other patients. It is, however, essential to mention that the lowest possible level of LDLc is preferred, according to the principle of “the lower the better”. Moreover, in patients with ASCVD who develop a second vascular event in 2 years (not necessarily of the same type as the first event), during a maximally tolerated treatment with statins, an LDLc target <1 mmol/L (40 mg/dL) can be taken into consideration [26,27,29].

From the secondary (co-primary) targets, a non-HDLc target of <2.2 mmol/L (85 mg/dL), <2.6 mmol/L (100 mg/dL) or <3.4 mmol/L (130 mg/dL) is recommended in patients with very high, high, or moderate CV risk. Regarding the apoB, the recommended targets are < 1.18 μmol/L (65 mg/dL), <1.46 μmol/L (80 mg/dL) or <1.82 μmol/L (100 mg/dL) for very high, high, or moderate CV risk. For TGs, it is recommended to achieve a target <1.69 mmol/L (150 mg/dL), while for the HDLc, a target of >1 mmol/L (40 mg/dL) in men and >1.29 mmol/L (50 mg/dL) in women should be aimed for. Some authors are also recommending the assessment of Lp(a), at least once, for an even more comprehensive overview of the CV risk, since it has been proven to have proatherogenic and procoagulant effects, while levels >1.07 μmol/L (30 mg/dL) and even more so, >1.78 μmol/L (50 mg/dL), are associated with an increased risk of developing a CV event. Nevertheless, more studies have to be performed in order to grasp the role of Lp(a)-lowering in reducing CV risk, independently of the lowering of the cholesterol found in other atherogenic particles, in patients with or without DM or prediabetes [26,27,29,30] (Table 1).

## 3. Management Strategies

### 3.1. Lifestyle Changes

For the treatment of lipid disorders, a comprehensive approach that includes lifestyle and pharmacological individualised interventions, taking into consideration the therapeutic targets and the characteristics of the patient, is recommended. The lifestyle changes include, alongside the diet, an increased, adapted, physical activity (usually with 3.5–7 h/week of moderate-intensity physical activity divided into 30–60 min on most days), smoking cessation and weight loss in patients who are overweight or obese [26].

The diet for preventing CVD in patients with dyslipidaemia should include a decreased intake of saturated fatty acids—the dietary factor with the highest impact on LDLc, alongside trans fatty acids—and an increased intake of wholegrain products, vegetables, fruit and fish. Moreover, a reduction in alcohol intake, a reduction in dietary carbohydrates—especially mono- and disaccharides—and a reduction of excessive body weight are shown to reduce the TG-rich lipoproteins, while the HDLc could be increased especially through more important physical activity. Nevertheless, recently, the focus has shifted from individual nutrients to dietary patterns such as the Mediterranean diet, which was associated with an improved metabolic control—including in patients with DM—in multiple trials such as PREDIMED (Prevencion con Dieta Mediterranea) or CORDIOPREV (Coronary Diet Intervention with Olive Oil and Cardiovascular Prevention); these are high-protein diets—taking into consideration the kidney function—that have shown a greater reduction in CV risk factors, including TG-rich lipoproteins and cholesterol levels in patients with DM, and with an avoidance of high-carbohydrate diets according to the PURE study. However, although n-3 fatty acid supplements are shown to decrease TG-rich lipoproteins, recent guidelines do not recommend them for secondary prevention of CVD in patients with T2DM [26,29].

Regarding the role of nutraceuticals in the management of lipid disorders, although they should not replace pharmacotherapy, some of the supplements—still under debate regarding their safety and efficacy—could help in lowering LDLc levels such as phytosterols, monacolin and red yeast rice [26,31,32].

### 3.2. Pharmacotherapy

The pharmacological treatment of lipid disorders includes, according to each case, the prescription of statins, bempedoic acid, and ezetimibe, agents that inhibit the action of proprotein convertase subtilisin/kexin type 9 (PCSK9), either as monoclonal antibodies or as small interfering RNA, alongside therapies that target the residual risk reduction and new therapies currently under trial or development.

#### 3.2.1. Statins

The first-line treatment in dyslipidaemias is represented by statins, pharmacologic agents; these not only lower significantly, in a dose-dependent manner, the level of LDLc, slightly lower the level of TGs and slightly increase the level of HDLc, but are also proven to have beneficial pleiotropic anti-inflammatory, antioxidant, antithrombotic effects and also to improve endothelial function, with the subsequent stabilization of atherosclerotic plaques and a reduction in CV mortality and morbidity. As a mechanism of action, statins inhibit the 3-hydroxy-3-methyl-glutaryl Coenzyme-A reductase (HMG-CoA reductase), with a consecutive decrease in the hepatic cholesterol biosynthesis that affects the intracellular level of cholesterol, with an up-regulation process in which the LDL receptors are involved and with the increased hepatic uptake of LDL particles, with a decrease in the plasmatic level of this lipoprotein [26,33].

The impact of reducing the LDLc levels depends on the intensity of treatment; therefore, a high-intensity statin therapy, which is represented by a dose of 40–80 mg of atorvastatin or 20–40 mg rosuvastatin, can lead to LDLc reductions of more than 50%; a moderate-intensity statin therapy, which is represented by a dose of 10–20 mg atorvastatin or 5–10 mg rosuvastatin, can lead to reductions of approximately 30–50%, while the low-intensity statin therapy is usually represented by other statins, with LDLc reductions of less than 30% [34].

Statin therapy, alongside different types of hypercholesterolemia, is recommended for the prevention of CVD. The contraindications of this class of drugs are the hypersensitivity to the product, pregnancy, and active chronic hepatic diseases or rhabdomyolysis, while the side effects, also described as statin intolerance, include an increase in transaminases or myopathy, ranging from myalgia and/or an increase in creatine kinase to exceptional cases of rhabdomyolysis, described in 1–3 cases/100,000 patient years. In cases where myopathy is present, depending on the intensity of symptoms, the level of creatine kinase or the presence of rhabdomyolysis, the temporary discontinuation, and replacement with another statin or another class of pharmacologic agent can be recommended [35,36].

Recent guidelines recommend the prescription of a high-intensity statin therapy up to the maximally tolerated or allowed dose for reaching the LDLc target, not only for the LDLc-reduction effect itself, but also for the effects in reducing CV mortality and morbidity that have been proven in multiple meta-analyses and landmark clinical trials such as ASCOT with atorvastatin, JUPITER with rosuvastatin or 4S with simvastatin [26]. Different subgroup analyses have shown protective effects of statins in patients with prediabetes and, although in this particular group of individuals with an increased risk of developing DM the association between newly-diagnosed cases of DM and statin therapy should be considered, the benefits almost always outweigh the diabetogenic risk [13]. In patients with DM at high or very high CV risk—which, in clinical practice, represent the majority of patients with DM—high-intensity statin therapy is recommended [29].

#### 3.2.2. Bempedoic Acid

In patients with statin intolerance, especially when considering statin-induced myopathy, bempedoic acid can be recommended as an alternative. Bempedoic acid, a pharmacologic agent that has been recently approved in the treatment of lipid disorders, a pro-drug which is activated in the liver by very long-chain acyl-CoA synthetase (ACSVL1), inhibits the adenosine triphosphate (ATP) citrate lyase and, consecutively, the hepatic biosynthesis of cholesterol. Since the active bempedoic acid is not found in the skeletal muscle, in clinical trials and other subsequent studies the adverse event rate was similar to that of placebo, even in patients with muscle symptoms while using statins. Regarding the LDLc-lowering effect, bempedoic acid was shown to lower it by approximately 30% in monotherapy and it was also associated with a significant decrease in the composites of CV death, non-fatal myocardial infarction, non-fatal stroke and coronary revascularization, while also not being associated with new-onset diabetes. However, these data have only been recently published and have not yet been included as recommendations for guidelines regarding the management of dyslipidaemia in patients with DM or prediabetes [26,29,37,38,39].

#### 3.2.3. Ezetimibe

In the case in which the LDLc target is not reached with a maximally tolerated or allowed dose of statin, the intensification of the lipid-lowering treatment with ezetimibe is recommended. This pharmacologic agent selectively inhibits the intestinal absorption of dietary and biliary cholesterol by more than 50%, which contributes to an LDLc reduction of approximately 20% in monotherapy and 65% when being administered in combination with a high-intensity statin therapy. The mechanism of action is represented by the inhibition of the transport protein Niemann-Pick C1-like 1 (NPC1L1), with the lowering of cholesterol absorption and, subsequently, with the reduction in the hepatic afflux of cholesterol, with an LDL receptor up-regulation and an increased uptake of these lipoproteins [40].

The contraindications and side effects are similar to those of statin therapy—ezetimibe is not recommended in cases of hypersensitivity to the product, pregnancy and active chronic hepatic diseases, while the side effects consist of myalgias, increases in creatine kinase or transaminases, gastrointestinal symptoms or headaches [26,41].

The beneficial effects of ezetimibe on CV mortality and morbidity have been proven in trials such as IMPROVE-IT (Improved Reduction of Outcomes: Vytorin Efficacy International Trial), in which ezetimibe has been associated with simvastatin in patients with a history of acute coronary syndrome. These results, alongside many others, have contributed to recommending ezetimibe in patients with DM as a second-line therapy after statins or in patients with statin intolerance, especially since in the DM subgroup the benefits have been even more important than in other subpopulations and since patients with type 1 DM appear to have an increased cholesterol absorption. Its effects on patients with prediabetes in comparison to those with DM or a normal glucose metabolism are not very well known yet [13,19,26,29].

#### 3.2.4. Pharmacologic Agents That Inhibit PCSK9

In the cases in which the LDLc therapeutic target is not reached even with a combination treatment with a statin and ezetimibe or if any of these pharmacologic agents cannot be administered, according to the most recent guidelines, the initiation of the treatment with agents that inhibit PCSK9 is recommended, either as a monoclonal antibody or an siRNA (small interfering RNA)-based technology.

PCSK9 is a protein which, in high concentrations or in cases when it has a more intense action, is associated with a reduction in the expression of LDL receptors through their increased catabolism and decreased recycling and with a consecutive reduction in LDL particle uptake and with plasmatic LDLc increase. The binding of monoclonal antibodies to PCSK9 will lead to a reduction in the plasmatic level of this protein, with increased recycling of LDL receptors and, thus, to a plasmatic LDLc reduction. The two drugs that are currently approved are alirocumab and evolocumab, and both contribute to a reduction in LDLc by approximately 60% in monotherapy and 85% in combination with a high-intensity statin therapy and ezetimibe, effects that lead to them being recommended in patients with familial hypercholesterolemia. Additionally, they have an effect in reducing Lp(a), unlike statins, which also contributes to their beneficial effects regarding CV morbidity and mortality, which have been proven in studies such as ODYSSEY Outcomes (Evaluation of Cardiovascular Outcomes After an Acute Coronary Syndrome During Treatment With Alirocumab) with alirocumab and FOURIER (the Further Cardiovascular Outcomes Research with PCSK9 Inhibition in Subjects with Elevated Risk) with evolocumab [42,43,44].

Regarding their usage in daily medical practice, the monoclonal antibodies that inhibit PCSK9 are administered as a subcutaneous injection, once every 2 weeks or every 4 weeks, and their reported side effects are itching at the site of injection or flu-like symptoms. The indications, both for alirocumab and evolocumab, are different types of hypercholesterolemia and cases where additional prevention of ASCVD is needed [26].

Different subgroup analyses have shown protective effects of PCSK9 inhibitors in patients with prediabetes, while for patients with DM, subgroup analyses of FOURIER and ODYSSEY Outcomes have shown a significant reduction in major adverse cardiovascular events, with additional results regarding the atherogenic lipid-lowering effects from the following studies: BANTING (Evaluation of Evolocumab Efficacy in Diabetic Adults with Hypercholesterolemia/Mixed Dyslipidemia), BERSON (Safety and Efficacy of Evolocumab in Combination With Statin Therapy in Adults with Diabetes and Hyperlipidemia or Mixed Dyslipidemia) and ODYSSEY DM-DYSLIPIDEMIA (Efficacy and Safety of Alirocumab Versus Usual Care on Top of Maximally Tolerated Statin Therapy in Patients With Type 2 Diabetes and Mixed Dyslipidemia). They are also not shown to be associated with new-onset diabetes [13,29].

The more recent option in inhibiting PCSK9, as previously mentioned, is the treatment with siRNA, which inhibits the protein’s synthesis through the catalytic degradation of the messenger RNA for PCSK9. The representative of this class, inclisiran, which is also administered as a subcutaneous injection, although once every 3 and then 6 months, has proven a dose-dependent LDLc, lowering up to 50%, according to the results of the ORION trials. The drug was approved by the European Medicines Agency (EMA) in 2020 and by the U.S. Food and Drug Administration (FDA) in 2021. However, this recent information—including the benefits in patients with DM that were noticed in ORION-10, ORION-11 and ORION-4—have not yet been yet included as recommendations for guidelines regarding the management of dyslipidaemia in patients with DM or prediabetes [26,45,46].

#### 3.2.5. Residual-Risk Reduction

Despite all of these evidence-based therapies that can be used in lipid disorders, patients with DM or prediabetes can have a residual CV risk caused, among other things, by an increased level of triglyceride-rich lipoproteins (which can be noticed through a high non-HDLc), an increased Lp(a), inflammation, an additional thrombotic risk or the glucose metabolism impairment itself, with the latter being addressed especially through the treatment with a glucagon-like peptide 1 receptor agonist (GLP-1 RA) and/or a sodium-glucose cotransporter-2 inhibitor (SGLT2i).

In patients with an increased level of triglyceride-rich lipoproteins, especially when TGs are >2.25 mmol/L (200 mg/dL), alongside lifestyle changes, the treatment with n-3 polyunsaturated fatty acids or fibrates can be recommended, and are interventions that have proven to bring additional CV benefits in multiple clinical trials [47]. In this regard, the REDUCE-IT (Reduction of Cardiovascular Events with Icosapent Ethyl—Intervention Trial) has shown a significant reduction in the risk of ischemic events, including the CV mortality risk, in patients previously treated with statins, with an increased cardiovascular risk and hypertriglyceridemia with the administration of icosapent ethyl in a high dose, a purified and stable version of eicosapentaenoic acid [48]. These effects have also been consistent in patients with DM. Moreover, the fibrates, agonists of the peroxisome proliferator-activated receptor-alpha (PPAR-α), which have proven efficacy in reducing the pre-prandial and postprandial level of plasmatic TG by approximately 50% and in producing a significant reduction in triglyceride-rich remnant lipoproteins and a slight increase (<20%) in HDLc, could have an impact on the reduction in CV risk, which is dependent on the reduction in non-HDLc. These results that have been shown in some studies such as the FIELD Study, ACCORD-Lipid, and PROMINENT, still need to be confirmed in future research, in patients with or without prediabetes or DM [26].

Regarding the reduction in the residual inflammatory risk, CANTOS (The Canakinumab Anti-inflammatory Thrombosis Outcome Study) with canakinumab, a monoclonal antibody against interleukin-1β, there has proven to be a significant reduction in recurring CV events, independent of the level of plasmatic lipids—yet this is not used in the treatment of dyslipidaemia, because of the adverse effects—while the LoDoCo2 (Low Dose Colchicine) and COLCOT (Colchicine Cardiovascular Outcome Trial) with colchicine, in patients with coronary heart disease and with a recent acute myocardial infarction, respectively, have also proven the reduction in CV events. These results have contributed to the inclusion of a class IIb level of evidence recommendation for using low-dose colchicine in the secondary prevention of CVD in the *2021 ESC Guidelines on cardiovascular disease prevention in clinical practice*, without specific recommendations for patients with DM or prediabetes [27,49,50,51].

### 3.3. Management Strategies in Patients with Diabetes or Prediabetes

Regarding the lipid-lowering treatment recommendations of the current guidelines, the *2023 ESC Guidelines for the management of cardiovascular disease in patients with diabetes* are recommending statins as the first-line drug in patients with DM in order to achieve the LDLc- or non-HDLc- target levels, according to their CV risk, usually a high-intensity statin prescribed up to the highest tolerated dose. If the LDLc target, which is set after the assessment of CV risk based on existing risk factors and comorbidities is not reached with statins, a combination therapy with ezetimibe is recommended (or a monotherapy with ezetimibe in cases of statin intolerance), while if the target is not reached even with a combination, a PCSK9 inhibitor is recommended, especially in patients with a very high CV risk, as a combination, or instead of statins, in cases of intolerance. In the case of hypertriglyceridemia, in patients with DM, high-dose icosapent ethyl (2 mg b.i.d.) may be considered (class IIb recommendation, level of evidence B), in combination with a statin, without mention of using fibrates [29]. Nevertheless, in severe hypertriglyceridemia, fibrates alongside n-3 polyunsaturated fatty acids are recommended in order to prevent an acute pancreatitis [34].

Similar recommendations regarding the LDLc lowering—with an additional emphasis on lifestyle changes, an intensification of statin therapy when possible and reminders than statins are not recommended in pre-menopausal female patients who are considering pregnancy or are not using adequate contraception—can be found in the *2019 ESC Guidelines for the management of dyslipidaemias*, in the *2023 Standards of Care in Diabetes* published by the American Diabetes Association, or in multiple position papers [25,26,52,53]. These recommendations are similar to those issued by the American Heart Association and the American College of Cardiology in their *2018 Guideline on the Management of Blood Cholesterol*, although the CV risk assessment and treatment targets are different from those of the ESC, with higher values in the former [34].

**Figure 1:**
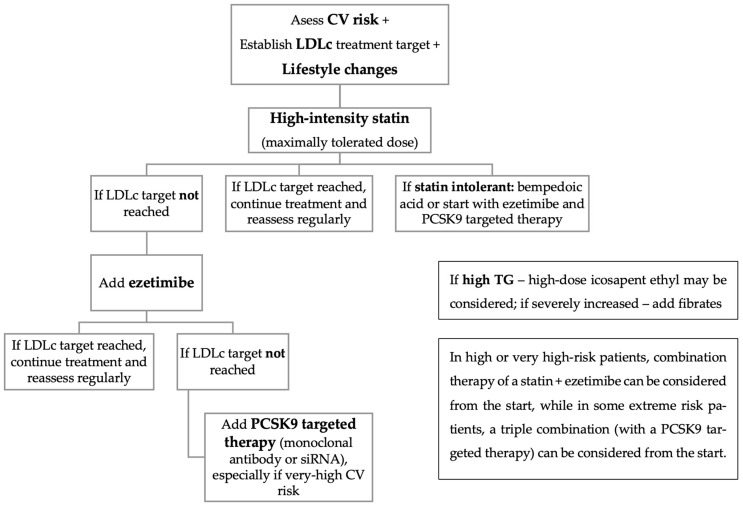
Lipid disorders management strategy in patients with DM.

Regarding high- or very high-risk patients, especially in the presence of CV disease, a combination therapy of a statin and ezetimibe can be considered as a first step, while in some extreme-risk patients a triple combination (with a PCSK9 targeted therapy) can be considered from the start [54]. Moreover, the International Lipid Expert Panel (ILEP) recommends the early usage of bempedoic acid in patients with partial or complete statin intolerance [55] (Figure 1). When taking into consideration patients with DM and chronic kidney disease (CKD), an association frequently encountered in clinical practice, the recommendations for a holistic approach are very similar, with statins being considered first-line drugs, either in a moderate- or a high-intensity therapy, with a possible exception in patients under chronic dialysis, a situation still evaluated in clinical trials [56,57].

However, for patients with prediabetes, there are currently no direct recommendations for managing lipid disorders. They could be treated similarly to those who have been diagnosed with type 2 DM, with a multifactorial, interdisciplinary approach, while addressing all CV risk factors, including hypertension or dysglycaemia, with pharmacologic agents with pleiotropic beneficial effects [26]. Nevertheless, the challenges of an early and correct diagnosis of prediabetes, the unawareness of this metabolic condition—only in five individuals with prediabetes are aware of this diagnosis—the lack of adequate inclusion or emphasis on this category of patients in clinical trials with evidence arising usually from post hoc analyses, and the difficulty in maintaining a rigorous follow-up, lead to insufficient data regarding the effect of lipid-lowering strategies in this specific subpopulation and to the necessity of additional trials that would be translated into guideline recommendations [58].

## 4. Future Perspectives

The development of new agents for the treatment of dyslipidaemia is, currently, one of the most dynamic topics of research. From statin therapy and combinations of statins with ezetimibe, to fibrates and icosapent ethyl—oral medication administered daily—to treatments administered monthly or twice a month, such as the monoclonal antibodies that inhibit PCSK9, and to siRNA that inhibits the synthesis of PCSK9 administered only twice a year, the research is heading towards vaccines that could be administered yearly or even towards gene therapy that could have lifelong effects. All of the above aim to reach and maintain the primary (LDLc), secondary or co-primary (non-HDLc and apoB), or, recently, Lp(a) targets [59].

New classes of pharmacologic agents with new and innovative mechanisms of action are explored, agents that could join the already existing ones that inhibit the biosynthesis of cholesterol, the intestinal absorption of cholesterol or PSCK9. Other recent drugs inhibit the synthesis of lipoproteins that contain apoB, with effects on reducing LDLc (lomitapid, which inhibits the microsomal triglyceride transfer protein, approved by the EMA and FDA for homozygous familial hypercholesterolemia cases or mipomersen, an antisense oligonucleotide which inhibits the messenger RNA of apoB_100_, only approved by the FDA for homozygous familial hypercholesterolemia cases), in reducing LDLc, Lp(a) and TG (evinacumab, an angiopoietin-like protein 3—ANGPTL3—antibody) in reducing TG levels (volanesorsen, pradigastat) or in increasing HDLc levels. However, multiple studies suggest that low HDLc levels are not a cause of ASCVD (such as the inhibitors of the cholesteryl ester transfer protein—CETP—dalcetrapib, evacetrapib, anacetrapib, and obicetrapib) [26]. Other research is focused on reducing ASCVD through reducing inflammation with interleukin-6 inhibitors such as ziltivekimab, while genetic targets are also being explored [60,61,62].

However, more and more spectacular research with results which describe, with an ever-increasing clarity, complex mechanisms at an intracellular level, open new perspectives on the pathogenesis of lipid disorders.

## 5. Summary and Conclusions

Lipid disorders are modifiable risk factors commonly associated with DM and prediabetes, with a major impact on the early development of ASCVD. Their approach should be individualised according to patients’ CV risk and comorbidities; this usually comprises a combination of lifestyle and pharmacologic interventions, while monitoring the LDLc values—the primary target—and non-HDLc and apoB as secondary (or co-primary) targets, alongside TG, HDLc and, if available, Lp(a). While in patients with DM the current guidelines’ recommendations for LDLc lowering, while preventing CV events, include a high-intensity statin therapy wherever possible, with multiple options of intensification or alternatives such as ezetimibe, bempedoic acid, and monoclonal antibodies that inhibit PCSK9 or siRNA such as inclisiran for the management of dyslipidaemia in patients with prediabetes, more future studies that would lead to specific recommendations are needed. Nevertheless, ongoing research that explores new pathways of action will also contribute significantly to diversifying the range of pharmacologic agents and, therefore, in achieving and maintaining the lipid targets, reducing the risk of CV events and achieving CV prevention in the population of patients with prediabetes or DM.

## Figures and Tables

**Table 1:** CV risk in patients with type 2 DM and lipid therapeutic targets.

	Very High CV Risk	High CV Risk	Moderate CV Risk
*Patients with type 2 DM with*	1. Established ASCVD *or* 2. Severe TOD: - eGFR < 45 mL/min/1.73 m^2^ or - eGFR 45–59 mL/min/1.73 m^2^ and microalbuminuria (UACR = 3–30 mg/mmol = 30–300 mg/g) or - macroalbuminuria (UACR > 30 mg/mmol = 300 mg/g) or - microvascular disease in at least three different sites *or* 3. SCORE2-Diabetes ≥ 20%	SCORE2-Diabetes: 10%–<20%	SCORE2-Diabetes: 5%–<10%
*LDLc target*	<1.4 mmol/L (55 mg/dL) and a reduction of at least 50% compared to the initial level (whichever is the lowest)	<1.8 mmol/L (70 mg/dL) and a reduction of at least 50% compared to the initial level (whichever is the lowest)	<2.6 mmol/L (100 mg/dL)
*Non-HDLc target*	<2.2 mmol/L (85 mg/dL)	<2.6 mmol/L (100 mg/dL)	<3.4 mmol/L (130 mg/dL)
*ApoB target*	<1.18 μmol/L (65 mg/dL)	<1.46 μmol/L (80 mg/dL)	<1.82 μmol/L (100 mg/dL)
*TG target*	<1.69 mmol/L (150 mg/dL)	<1.69 mmol/L (150 mg/dL)	<1.69 mmol/L (150 mg/dL)
*HDLc target*	>1.29 mmol/L (50 mg/dL) (women) and >1 mmol/L (40 mg/dL) (men)	>1.29 mmol/L (50 mg/dL) (women) and >1 mmol/L (40 mg/dL) (men)	>1.29 mmol/L (50 mg/dL) (women) and >1 mmol/L (40 mg/dL) (men)

Adapted from the *2021 ESC Guidelines on cardiovascular disease prevention in clinical practice* and the *2019 ESC Guidelines for the management of dyslipidaemias*.
